# Rare Case of Necrotizing Fasciitis With Polyarteritis Nodosa in a Multi‐Comorbid Patient

**DOI:** 10.1002/ccr3.73624

**Published:** 2026-09-25

**Authors:** Syed Muhammed Salman Hassan, Sarim Hassan Shahab, Masroor Raza, Salman Saleem, Muhammad Fahad Hussain, Fazal Habib

**Affiliations:** ^1^ Nishtar Medical University Multan Pakistan; ^2^ Ch Pervaiz Elahi Institute of Cardiology Multan Pakistan; ^3^ Multan Medical & Dental College Multan Pakistan; ^4^ Sher‐e‐Bangla Medical College Barisal Bangladesh

**Keywords:** clinical bacteriology, LRINEC score, necrotizing fasciitis, surgical and medical management

## Abstract

Necrotizing fasciitis can mimic cellulitis in immunocompromised patients, delaying diagnosis. This case highlights the importance of early clinical suspicion despite low LRINEC score. Prompt surgical intervention, appropriate antibiotics, and recognition of underlying conditions like polyarteritis nodosa are critical to reduce morbidity and mortality in rapidly progressive soft tissue infections.

## 1 Introduction

Widespread fascial plane necrosis characterizes necrotizing fasciitis (NF), a severe, rapidly advancing soft tissue infection. It mostly affects skin and subcutaneous tissues and can cause systemic toxicity and multi‐organ failure if untreated [1]. Diabetes, immunocompromised states, trauma, intravenous drug use, vascular disease, obesity, skin infections, age extremes, and preexisting conditions like cellulitis are risk factors for necrotizing fasciitis [2]. The most common pathogens are Group A Streptococcus (GAS) and *Staphylococcus aureus* , including MRSA [1].

Polyarteritis nodosa (PAN) is a necrotizing vasculitis affecting medium‐sized and small arteries and can cause tissue ischemia and necrosis because of vascular inflammation and occlusion [3].

This life‐threatening illness requires quick diagnosis and treatment. This article emphasizes the importance of following NF treatment guidelines, including rapid diagnosis, effective antimicrobial therapy, and surgical intervention. These treatments improve patient survival and reduce complications.

## 2 Case History/Examination

**FIGURE 1:**
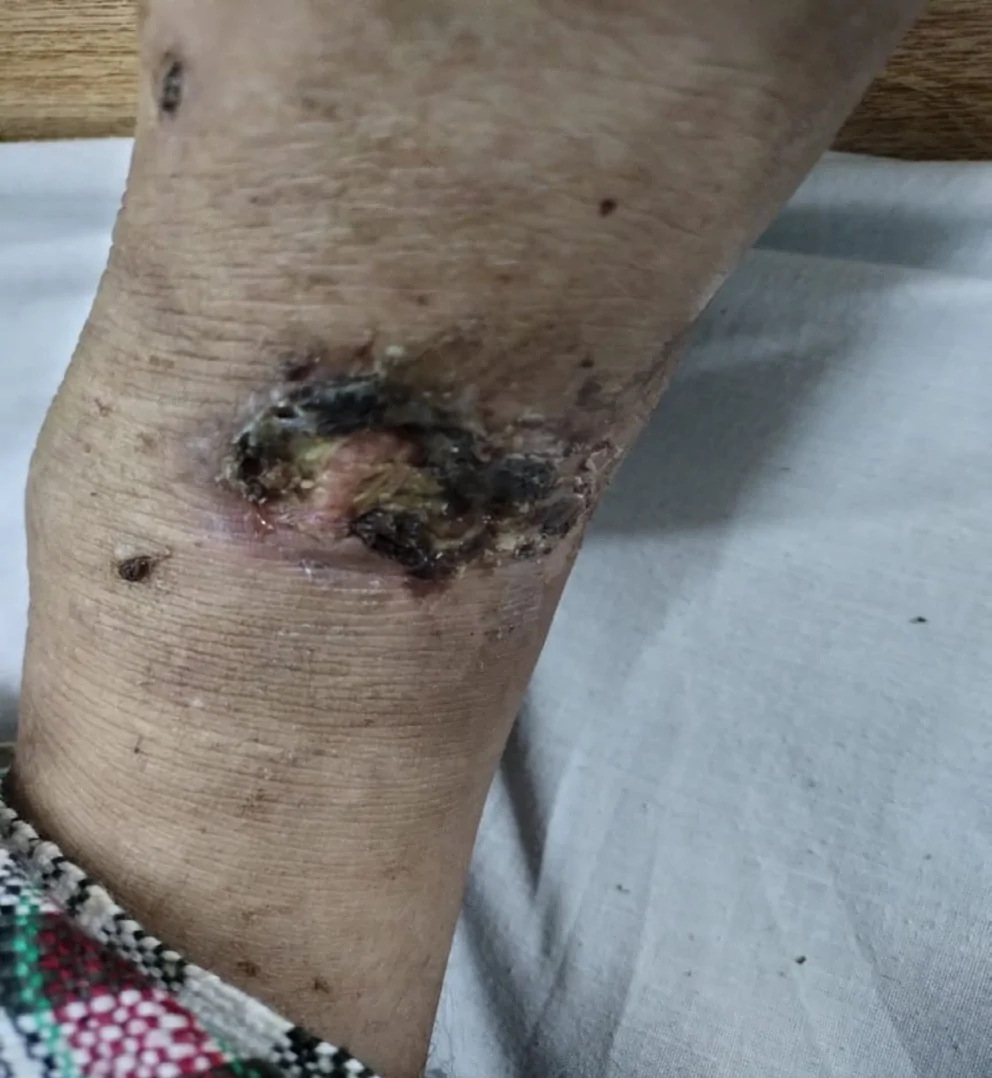
PAN associated ulcer 1.

**FIGURE 2:**
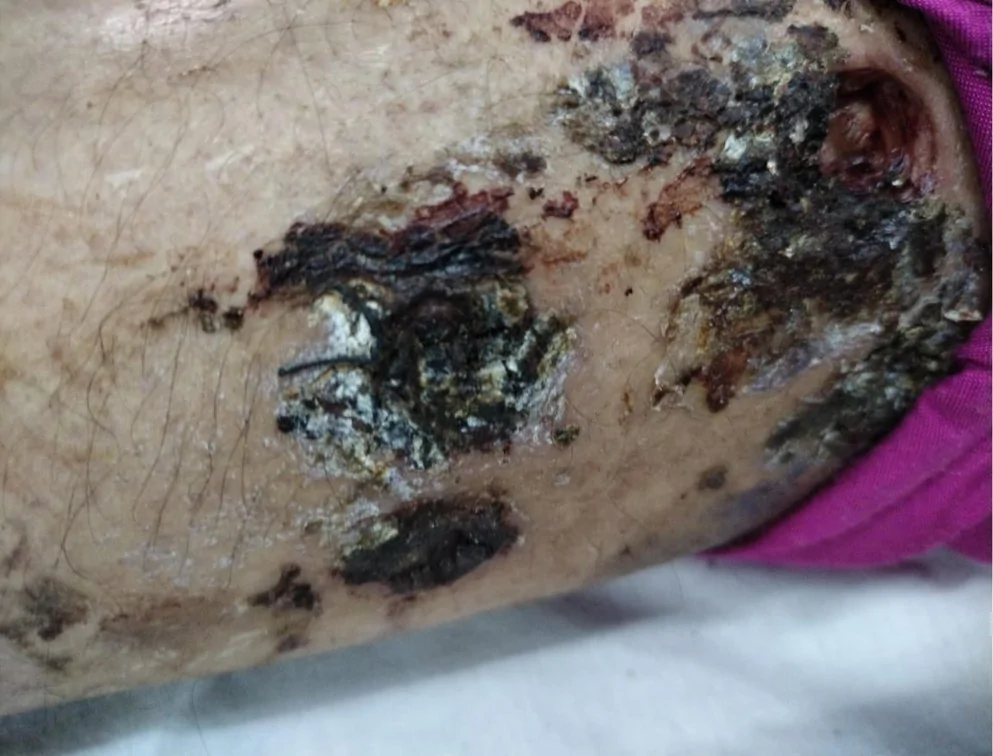
PAN associated ulcer 2.

A 38‐year‐old woman with treated Hepatitis C and polyarteritis nodosa (PAN) had significant burning pain, redness, and swelling in her right 3rd and 4th digits of the hands. The history revealed that she had recurring necrotic, weeping sores on her feet, thighs, arms, and torso for a month. After 14 days of vancomycin and sulzone therapy, her ulcers did not improve. An earlier histopathology evaluation revealed biopsy‐proven vasculitis, characterized by prominent involvement of small and medium‐sized vessels with acute inflammation and fibrinoid necrosis. No granulomas or cancers were found. These results revealed vasculitis, possibly caused by PAN, which was worsened by diffuse pyoderma gangrenosum and necrotic wounds (Figures 1 and 2). The skin lesions shown in Figures 1 and 2 were part of the patient's current illness and represented the recurrent necrotic and weeping lesions that had been present during the preceding month.

### 2.1 Clinical Examination

**FIGURE 3:**
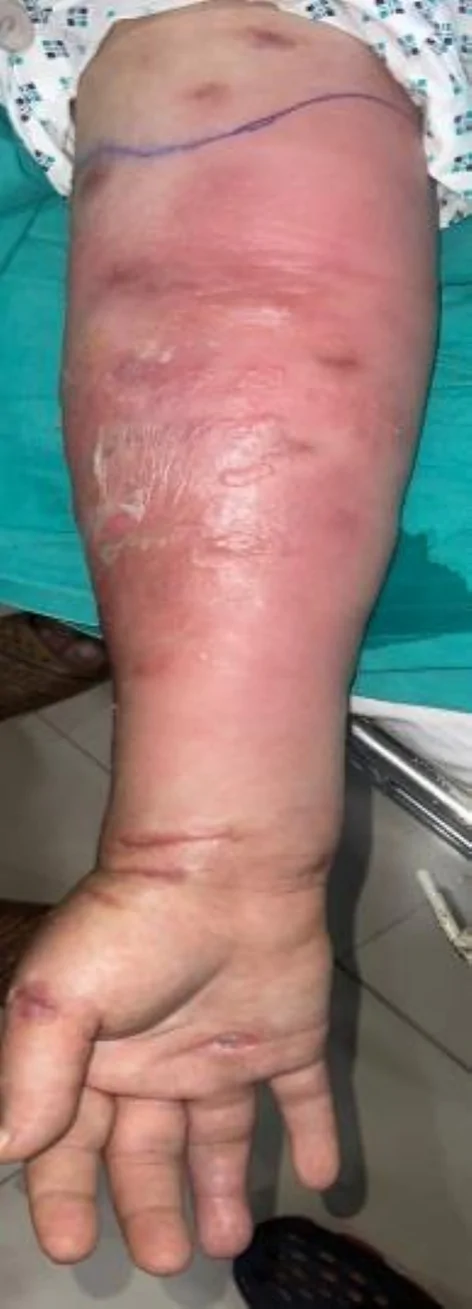
Picture showing spread of infection.

Over four days, her symptoms spread to the right hand and forearm. Erythema, swelling, pain, skin discolouration, fluid‐filled bullae, and necrosis covered the right hand and forearm upon examination. Necrotizing fasciitis was suspected due to palm ulcers leaking pus (Figure 3).

## 3 Differential Diagnosis, Investigations & Treatment

Given the acute and rapidly progressive presentation, chronic dermatologic conditions such as hypopigmented mycosis fungoides were considered less likely. The differential diagnosis for the rapidly developing pain, swelling, erythema, bullae formation, and necrosis of the right hand and forearm included severe cellulitis, necrotizing fasciitis, pyoderma gangrenosum flare in the setting of polyarteritis nodosa, compartment syndrome, and clostridial myonecrosis (gas gangrene). Compartment syndrome was initially considered because of the marked swelling and pain of the affected limb; however, the subsequent clinical course and operative findings favored a necrotizing soft tissue infection. Severe cellulitis was considered, but the disproportionate pain, rapid tissue destruction, bullae, and intraoperative findings favored the diagnosis of necrotizing fasciitis. The severe acute onset, purulent material, systemic inflammatory reaction, and prompt clinical improvement of the lesion after emergency surgical debridement and administration of a broad spectrum antibiotic led to a consideration of a superimposed necrotizing soft tissue infection. The clinical presentation of crepitus and imaging were not suggestive of the presence of gas in the soft tissue, and gas gangrene was considered less likely.

Ultrasound demonstrated findings supportive of necrotizing fasciitis, while the diagnosis was established clinically and confirmed during surgical exploration (Table 1).

**TABLE 1:** Summary of investigations.

Investigation	Result
ESR (Erythrocyte sedimentation rate)	59 mm/h
White blood cell count (WBC)	The initial leukocytosis (WBC 28.8 × 10^9^/L) decreased following surgical intervention and antimicrobial therapy, consistent with clinical improvement.
C3 and C4 (Complement levels)	Normal
CRP (C‐reactive protein)	CRP largely remained normal on laboratory studies.
Blood culture	No bacterial growth after 5 days (using Versa‐TREK system)
Skin biopsy	Prominent vasculitis involving small and medium‐sized vessels
GMS/PAS stain	Negative
Immunofluorescence studies	IgG, IgA, IgM, C1q, C3: Negative
Doppler ultrasound	Subcutaneous oedema in the affected limb

Immunosuppressive medications such as monthly cyclophosphamide injections and arthritis medication methylprednisolone may have caused this severe soft tissue infection. Because of the marked limb swelling and concern for compartment pressure, an emergency fasciotomy was initially performed to relieve tissue pressure. This was followed by surgical debridement of the devitalized tissue, which was performed once. The combination of urgent surgical intervention and antimicrobial therapy was used to control the rapidly progressive infection.

Postoperatively, negative‐pressure wound therapy (VAC) was instituted to promote wound healing and facilitate granulation tissue formation. The exact number of VAC dressing changes could not be determined because this information was not documented in the available medical records and was not recalled by the patient. Broad‐spectrum intravenous meropenem and levofloxacin were continued, while postoperative pain was managed with intravenous tramadol. Once healthy granulation tissue had developed and the infection was adequately controlled, the wound was closed by secondary closure without the need for skin grafting. She also had vaginal thrush and was given clotrimazole for it. Her chronic illness was confirmed by previous histopathological and microbiological studies. Previous skin biopsy results showed PAN vasculitis, excluding out infections and cancer. Even though the blood culture was sterile, clinical and imaging evidence strongly suggested NF, a probable PAN consequence. The patient improved significantly and exhibited no infection at her 10‐day follow‐up. This case shows that immunocompromised individuals with severe soft tissue infections underlying vascular disease benefit from histological evidence, early clinical diagnosis, vigorous surgical care, and targeted antimicrobial medication.

## 4 Results and Conclusion

The patient showed progressive clinical improvement following surgical debridement, negative‐pressure wound therapy, and antimicrobial treatment. She remained clinically stable throughout her postoperative course and was discharged after a 5‐day hospital stay with instructions for continued wound care. Complete wound healing was achieved within approximately three weeks, with no evidence of recurrent infection at follow‐up.

Laboratory and imaging can help diagnose NF after disproportional discomfort and fast swelling. Early identification, surgical debridement, and antibiotic therapy can save a patient's life are essential to reduce morbidity and mortality in rapidly progressive necrotizing fasciitis.

## 5 Discussion

A female patient with Polyarteritis Nodosa (PAN) initially had cellulitis symptoms. Her early symptoms included intermittent unexplained fever, discomfort, and right‐hand erythema. Nonspecific symptoms slowed diagnosis. The swelling spread to the right forearm, with fluid‐filled bullae and skin discolouration and necrosis. The diagnosis of necrotizing fasciitis was primarily clinical and was supported by laboratory findings, Doppler ultrasound, and confirmation during surgical exploration. Given the rapidly progressive clinical deterioration, surgical exploration was undertaken without delaying definitive management for advanced imaging.

Several predisposing variables were found. Long‐term corticosteroid medication (prednisolone) for active arthritis reduced her immune system, rendering her more susceptible to infections. In addition, she had treated Hepatitis C and had cyclophosphamide, a strong immunosuppressant, for PAN. These causes damaged her immune system and advanced NF. Due to vasculitis, her peripheral veins were damaged; thus, drugs were given through a central venous catheter (CVC) to ensure good delivery.

Necrotizing Fasciitis (NF) causes significant inflammation and tissue necrosis, causing sharp pain, swelling, tenderness, and fever. In this case, LRINEC of 3 did not indicate NF, but elevated ESR, WBCs, clinical picture, and Doppler Ultrasound subcutaneous oedema required prompt therapy. Interestingly, the CRP remained largely within the normal range during the available laboratory assessments despite the severity of the infection. This highlights that individual inflammatory markers may be nondiagnostic and that necrotizing fasciitis should not be excluded solely on the basis of a normal CRP or a low LRINEC score when the clinical presentation is concerning. In this patient, the rapidly progressive local findings, marked leukocytosis, and operative findings supported the diagnosis and prompted urgent surgical management.

As in this patient, large bullae with bloody or yellowish fluid occur, although blackened necrotic lesions, which are typical of NF, are not usually present. Another hallmark is skin darkening. Untreated infection can spread to deeper tissues, including nerves, causing nerve damage and reduced pain or sensation. NF might be monomicrobial or polymicrobial. Most often implicated is Group A Streptococcus, followed by *Staphylococcus aureus* , including MRSA [1, 2]. Blisters with gas development suggest Clostridium species. Skin and blood cultures are critical for pathogen identification, although they may fail [3]. Rare bacterial necrotizing fasciitis causes fast tissue damage and high death. Necrotizing fasciitis is a rare infection with an estimated incidence of approximately 1–2 cases per 100,000 person‐years, although reported rates vary geographically [4, 5]. Early detection and active treatment of NF are crucial, especially in immunocompromised patients with susceptible diseases like PAN.

Patient's vasculitis, immunosuppression from corticosteroids for active arthritis, and earlier cyclophosphamide therapy were the main factors. These characteristics formed an ideal setting for NF development, emphasizing the need for close monitoring in high‐risk populations.

The gold standard is surgical debridement. Surgical debridement is the cornerstone of treatment for necrotizing fasciitis, and repeated debridement may be required in complex cases. To avoid sepsis, use bacteria‐specific antibacterials. Emergency fasciotomy was performed. Meropenem, Levofloxacin, and serrati peptidase were given for 7 days following surgery with enoxaparin to prevent thrombosis. Early identification, fasciotomy, and antibiotics were associated with clinical improvement.

### 5.1 Limitations

This case report has multiple limitations. First, the tissue culture and susceptibility testing of the wounds were not available, thus a microbiological confirmation of the causative pathogen was not possible and culture‐guided antimicrobial therapy was not available. As a result, diagnosis was limited to clinical means, and empiric broad‐spectrum antibiotics were administered. Second, advanced cross‐sectional imaging (computed tomography [CT] or magnetic resonance imaging [MRI]) to better delineate the extent of fascial involvement was not performed. A diagnosis was made largely by clinical means, using additional laboratory and Doppler ultrasound studies—and in a patient with such rapid clinical deterioration, prompt surgical action was deemed essential to prevent delay to definitive treatment. This is despite these limitations, with good clinical response to prompt surgical intervention and antimicrobial therapy suggesting the accuracy of the clinical diagnosis.

## Author Contributions

**Syed Muhammed Salman Hassan:** writing – original draft, conceptualization, writing – review and editing. **Sarim Hassan Shahab:** writing – review and editing, writing – original draft. **Masroor Raza:** writing – original draft, writing – review and editing. **Salman Saleem:** writing – original draft, writing – review and editing. **Muhammad Fahad Hussain:** writing – original draft, writing – review and editing. **Fazal Habib:** writing – review and editing, supervision.

## Funding

The authors have nothing to report.

## Ethics Statement

Ethical approval was taken verbally in consent.

## Consent

Written informed consent from the patient was obtained for publication according to journal guidelines.

## Conflicts of Interest

The authors declare no conflicts of interest.

## Data Availability

Data sharing not applicable to this article as no datasets were generated or analysed during the current study.
